# Peripheric facial paralysis as initial manifestation of occult metastatic neoplasia

**DOI:** 10.1016/S1808-8694(15)30668-6

**Published:** 2015-10-19

**Authors:** Fabio Brodskyn, Fernando Kaoru Yonamine, Olivia Capela Grimaldi Oliveira, Marcelo Ferreira dos Anjos, Norma de Oliveira Penido

**Affiliations:** 1 Resident physician; 2 Resident physician; 3 Resident physician; 4 Otorhinolaryngologist, Volunteer Physician – ENT Department - UNIFESP; 5 PhD. Affiliated professor – ENT Department - UNIFESP. Escola Paulista de Medicina - Universidade Federal de São Paulo

**Keywords:** metastasis, neoplasms, unknown primary

## INTRODUCTION

Peripheral facial paralysis is a relative common condition; it is mostly idiopathic, when it receives the name Bell's palsy. However, we should be aware of the differential diagnosis with less common severe or potentially lethal diseases.

Thus, there are cases in which facial paralysis is a manifestation of a temporal bone metastasis. This is a rare situation, although its incidence has apparently increased in recent years; its diagnosis may be delayed by lack of appropriate and prompt investigation by a specialist.^1^

In this article we report a case of a patient with peripheral facial paralysis as the first manifestation of a metastasis originating from a hidden tumor; we also review the literature on the case. We highlight this case due to the difficulty in making the diagnosis and its unfavorable outcome.

## CASE REPORT

SMS, a female white patient aged 40 years, arrived at an emergency otorhinolaryngology clinic with a complaint of right peripheral facial paralysis that had started 15 days ago; there had been no previous otorrhea or otalgia. The personal and family history added no further information.

The physical examination showed that the patient was in good general health; there was grade IV facial paralysis.^2^ Otoscopy revealed right tympanic membrane retraction and a whitish tumor on the attical region with lamellae (suspected cholesteatoma).

**Figure 1:**
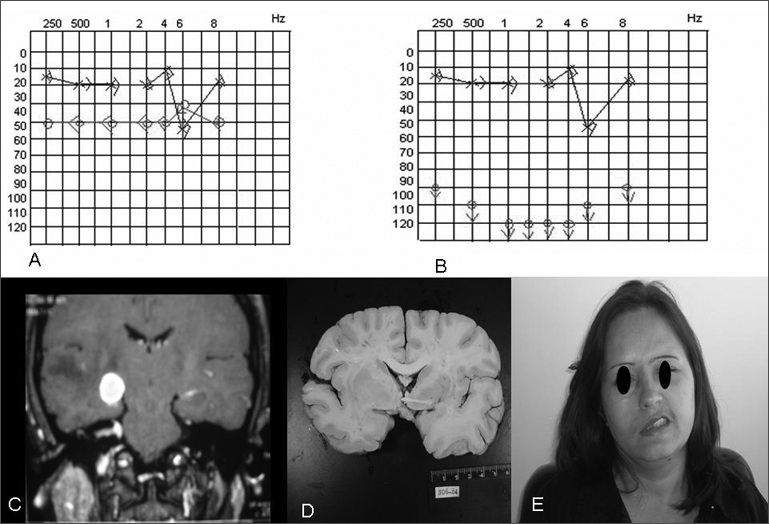
A - initial audio; B - final audio; C - MRI with a metastasis on the MAI; D - necropsy result; E - facial paralysis G V to the left.

Computed tomography of the temporal bones and audiometry (Fig. 1A) were done initially; the patient was monitored weekly in the outpatient unit.

One week later the patient complained of hypoacusis; the facial paralysis had worsened to grade V (Fig. 1E). A second audiometry (Fig. 1B) confirmed the poorer thresholds, progressing to profound right dysacusis. Electroneuromyography revealed signs of a chronic severely compromised right facial nerve, of an axonal nature. With the worsened clinical picture, associated with rapidly progressive hearing loss, which are uncommon findings in Bell's palsy, the patient underwent magnetic resonance imaging (Fig. 1C). This exam showed innumerable images in the cerebral parenchyma associated with significant perilesional edema; the images suggested metastases.

The patient was admitted into hospital to search for the primary site. Breast, lung, gastrointestinal or skin tumors were discarded. Before locating the primary tumor, the patient died of intracranial hypertension notwithstanding therapy, two months after facial paralysis had started.

Necropsy (Fig. 1D) did not reveal any macroscopic primary tumor. Immunohistochemical studies of metastases suggested a pancreatic cancer; the final diagnosis, however, was undifferentiated carcinoma of indeterminate origin.

## DISCUSSION

Our review of the literature brought up 148 published cases of temporal bone lesions as the first manifestation of metastases. The main primary sites were, in decreasing order, breast, lung, kidney, stomach, and prostate cancers.^3^ The main symptoms are facial paralysis, hypoacusis, tinnitus, altered bodily balance, and vertigo; these symptoms may be isolated or associated.^1^,^3^,^4^

Cases of facial paralysis secondary to malignances generally have a poor prognosis; the paralysis progresses and does not regress with any therapy, which is uncommon in Bell's palsy cases.^1^,^3^,^4^ The etiological diagnosis may be delayed, which worsens the prognosis. Exams are mandatory in such cases, especially magnetic resonance imaging.^3^,^4^

## FINAL COMMENTS

We should always be watchful in all cases of facial paralysis, especially those with an atypical progression, so that the true diagnosis is not delayed. We also concluded that whenever the presentation is atypical, or if any of the initial exams are not aligned with the clinical history, image exams become of great use and should not be postponed.

## References

[1] Miro Castillo N, Roca-Ribas Serda F, Barnadas Molins A, Prades Marti J, Casamitjana Claramunt F, Perello Scherdel E (2000). Facial paralysis of Metastatic Origin. Review of metastatic lesions of the temporal bone. An Otorrinolaringol Ibero Am..

[2] House JW, Brackmann DE (1985). Facial nerve grading system. Otolaryngol Head Neck Surg..

[3] Boahene DO, Olsen KD, Driscoll C, Lewis JE, McDonald TJ (2004). Facial nerve paralysis secondary to occult malignant neoplasms. Otolaryngol Head Neck Surg..

[4] Alaani A, Hogg R, Saravanappa N, Irving RM (2005). An Analysis of diagnostic delay in unilateral facial paralysis. J Laryngol Otol..

